# Validation of a new portable system containing both FeNO analysis and spirometry measurement

**DOI:** 10.3389/fmed.2023.1210329

**Published:** 2023-08-31

**Authors:** Yong Li, Ke Huang, Wei Li, Yaodie Peng, Xingyao Tang, Ting Yang

**Affiliations:** ^1^National Center for Respiratory Diseases, State Key Laboratory of Respiratory Health and Multimorbidity, National Clinical Research Center for Respiratory Diseases, Institute of Respiratory Medicine, Chinese Academy of Medical Sciences, Department of Pulmonary and Critical Care Medicine, Center of Respiratory Medicine, China-Japan Friendship Hospitall, Beijing, China; ^2^Peking University China-Japan Friendship School of Clinical Medicine, Beijing, China; ^3^Capital Medical University, Beijing, China

**Keywords:** FeNO, spirometry, portable system, airway disease, asthma

## Abstract

**Introduction:**

Pulmonary function tests and FeNO measurements are widely used for the diagnosis and management of respiratory diseases. They are used to evaluate airway limitation and respiratory inflammation. Standard spirometers and nitric oxide (NO) analyzers are widely used in hospitals. However, their high price has made some hospitals in underdeveloped areas unable to afford or purchase these devices. The development of a new portable system (SUNVOU TM2125) combining FeNO measurement and spirometry provides additional possibilities for optimizing the diagnosis and management of respiratory diseases. However, its accuracy needs further validation.

**Methods:**

The FeNO analysis component of SUNVOU TM2125 was compared with that of a widely used NO analyzer (NIOX VERO). The spirometry component of the TM2125 was compared with a standard spirometer (Jaeger MasterScreen) for pulmonary parameters such as FEV1, FVC, FEV1/FVC, and PEF. Pearson correlation and Bland–Altman plots were used to evaluate the agreement between the devices.

**Results:**

FeNO values measured using TM2125 were higher than those measured using VERO, with a mean difference of 1.8 ppb. There was a strong correlation between FeNO values measured using the two devices (*r* = 0.988, *p* < 0.001). Bland–Altman plots showed a high degree of agreement between the two devices, with 93.3% of values within the 95% confidence interval range. The spirometric parameters (FEV1, FVC, FEV1/FVC, and PEF) measured using the TM2125 were lower than those measured using the MasterScreen. Good correlations were observed between the values measured using the TM2125 and MasterScreen (*r* > 0.9). Based on the Bland–Altman plots, there was a high degree of agreement between the devices.

**Conclusion:**

The accuracy of FeNO and spirometry measurements using SUNVOU TM2125 was validated. This can help improve the diagnosis and monitoring of chronic respiratory diseases in underdeveloped countries.

## 1. Introduction

The measurement of exhaled NO and pulmonary function is an important examination tool in the respiratory department. They are recommended by the Global Initiative of Asthma (GINA) and the National Institute for Health and Care Excellence (NICE) for the diagnosis and management of asthma (1, 2). FeNO is recommended for diagnosing eosinophilic airway inflammation, determining the likelihood of steroid responsiveness, and predicting the risk of exacerbations (3, 4). Pulmonary function tests can be used to evaluate airflow limitation, disease severity, and control levels (1, 5). Airway inflammation and airflow limitation are two treatable traits of airway disease and require precision management.

Pulmonary function alone cannot provide full evaluation in asthma patients; however, combining FENO and pulmonary function together can provide more diagnostic and therapeutic information for clinicians. FeNO measurements combined with pulmonary function tests play an important role in the diagnosis of chronic airway disease, prediction of treatment response, and guidance of treatment plans. They can help clinicians evaluate the disease more comprehensively and systematically (6, 7). However, due to the heterogeneity of asthma patients and variability in the test results, clinicians cannot rely only on FENO to guide care (8). At present, medical institutions require both an exhaled NO analyzer and a pulmonary function analyzer to carry out the above two tests. For many hospitals, it may be difficult to afford the two equipment, especially the pulmonary function analyzer. To solve this problem, a new type of equipment (SUNVOU-TM2125) was developed that combines an NO analyzer and a spirometer. Using SUNVOU-TM2125, clinicians can simultaneously measure FeNO values and important spirometer parameters, such as FEV1, FVC, and PEF. This is of great significance for underdeveloped areas.

This study was designed to test the ability of SUNVOU-TM2125 to accurately measure FeNO levels and pulmonary parameters (FEV1, FVC, FEV1/FVC%, and PEF). The spirometry component of SUNVOU-TM2125 was compared with a standard laboratory spirometer (MasterScreen, Jaeger, Germany) for pulmonary parameters, and the NO analysis component was compared with a widely used NO analyzer (NIOX VERO, Aerocrine, Sweden) for FeNO values.

## 2. Materials and methods

### 2.1. Study population

We recruited adolescent patients who visited the Pulmonary and Critical Care Department of the China-Japan Friendship Hospital between September 2022 and December 2022. Patients who were unable to undergo FeNO testing or spirometry were excluded. This study was approved by the China-Japan Friendship Hospital Review Board (2022-KY-141). All the participants provided written informed consent.

### 2.2. FeNO measurements

According to the product manual, NIOX VERO is a service-and calibration-free system. SUNVOU-TM2125 was calibrated monthly using a calibration gas with an NO concentration of 60 ppb. The standard error meets the technical requirements of the product (error < 3 ppb or < 10%). NIOX VERO cannot be calibrated with a standard gas. The test environment (temperature, atmospheric pressure, humidity, etc.) was the same for all subjects.

FeNO measurements were performed strictly according to the technical standards published by ATS and ERS using SUNVOU-TM2125 and NIOX VERO in random order (9). Before the test, the operator explained the procedures to the participants, consulted and filled in their basic information, and ensured that they fully understood the test method. During the test, patients sat and covered their mouths with a filter. After deep inhalation through the mouth to total lung capacity (TLC), they were asked to exhale through the mouthpiece at a constant flow rate of 50 mL/s for 10s. The FeNO test results were displayed after approximately 1 min.

### 2.3. Spirometry measurements

TM2125 and MasterScreen were calibrated using standard 3-L calibration syringes. The measured volume should meet the accuracy requirement of ±3.5% (10). The preheating time of the two devices was 15 min, and the test environment (temperature, atmospheric pressure, humidity, etc.) was consistent. The participants underwent spirometry using TM2125 and MasterScreen, with a trained technician guiding the procedure. During the measurement, the participants were seated and their noses were clipped to avoid air leakage. They inhaled deeply to full lung capacity and then exhaled without hesitation in a burst of force. The exhalation time should be more than 6 s. The participants were asked to repeat the measurement three times to get three acceptable results. The difference between the best two acceptable FEV1 and FVC should be less than 0.15 L. The results of FEV1, FVC, FEV1/FVC, and PEF were recorded. FEV1 and FVC had the largest values among the three results. The PEF was chosen from the best curve.

### 2.4. Statistical analysis

Statistical analyses were performed with SPSS version 22.0. Data were presented as mean ± SD. Differences between the values measured by the two devices were analyzed using a paired *t*-test. The correlation and agreement between the pulmonary function parameters measured by the devices were assessed using Pearson’s correlation coefficient and Interclass Correlation Coefficient (ICC). An ICC greater than 0.8 indicated strong consistency. Bland–Altman plots were used to evaluate the agreement between the devices using MedCalc version 18.2.1. Statistical significance was defined as *p* < 0.05.

## 3. Results

### 3.1. Participants

A total of 301 participants took the spirometry measurements using SUNVOU-TM2125 and MasterScreen Diffusion. A total of 148 adolescent patients participated in the FeNO comparison test using SUNVOU-TM2125 and NIOX VERO. The demographic information of the study population is summarized in Table 1.

**Table 1:** Subject demographics.

	FeNO measurements	Spirometry measurements
Number	148	301
Age, years	43.8 ± 15.1	59.2 ± 12.1
Sex (male/female)	82/66	205/96
Smoking(*n*, %)	35 (23.6)	160 (53.2)
Disease, *n*
Asthma	58	45
Chronic cough	23	19
COPD	2	66
Allergic rhinitis	9	1
Others	56	170

### 3.2. FeNO measurements

A total of 148 patients took FeNO measurements, with an average age of 43.8 ± 15.1 years. The male: female ratio was 82:66. The medical histories of all patients were recorded, of which 58 had asthma, 23 had chronic cough, two had COPD, nine had allergic rhinitis, and 56 had other conditions. Of these patients, 35 (23.6%) were smokers (Table 1).

**Figure 1:**
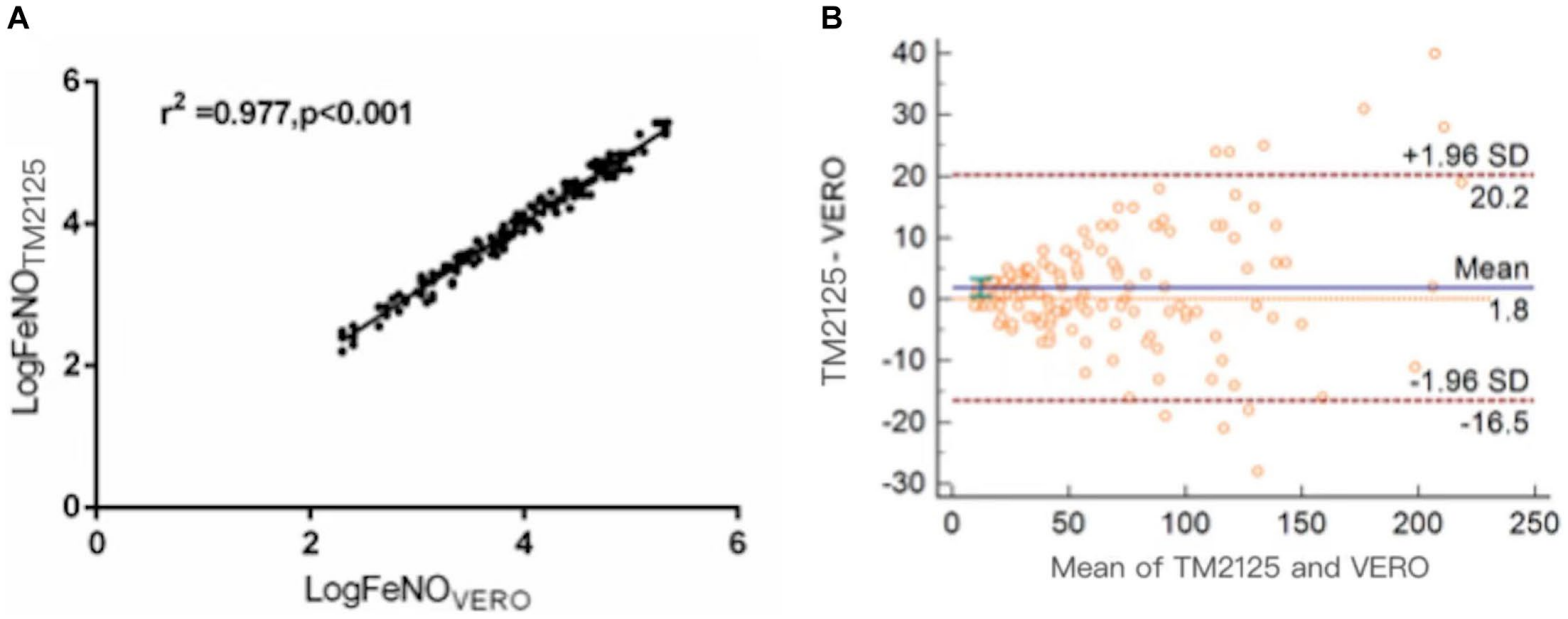
Correlation and agreement between FeNO values measured by TM2125 and VERO. **(A)** There was a positive correlation between FeNO values measured by TM2125 and VERO; **(B)** Bland–Altman plot showed a high degree of agreement between FeNO values measured by TM2125 and VERO.

FeNO measured using TM2125 was higher than that measured using VERO (*p* = 0.015). There was a strong positive correlation between FeNO values measured using both devices (Spearman’s correlation coefficient: *r* = 0.988, *p* < 0.001; Figure 1A). The conversion equation was calculated using linear regression analysis as follows: $\log FeNO_{CA2122}=0.502+0.883\times \log FeNO_{VERO.}$

The Bland–Altman plot showed a moderate degree of agreement, with a mean difference of 1.8 ppb (95% CI: −16.5 ~ 20.2; Figure 1B). A total of 6.7% (10/148) of the values were outside the 95% CI. Within the 95% CI, the absolute value of the difference was up to 19 ppb (TM2125: 228 ppb, VERO: 209 ppb), 8.7% of the average value, which is within tolerance limits. According to the official ATS clinical practice guidelines, a clinically significant change in FeNO is >10 ppb (or 20%). So device differences within these limits were considered clinically non-significant.

### 3.3. Spirometry measurements

**Figure 2:**
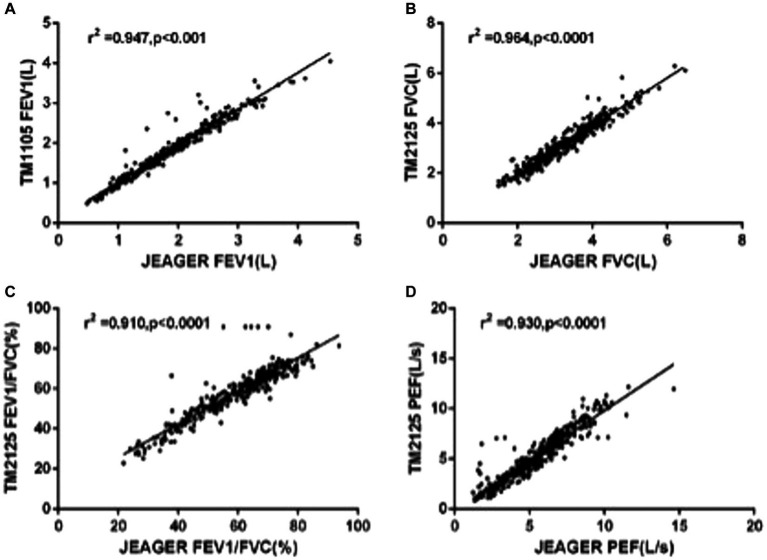
Linear regression with Pearson correlation analysis of spirometric parameters: **(A)** FEV1, **(B)** FVC, **(C)** FEV1/FVC%, and **(D)** PFF.

**Figure 3:**
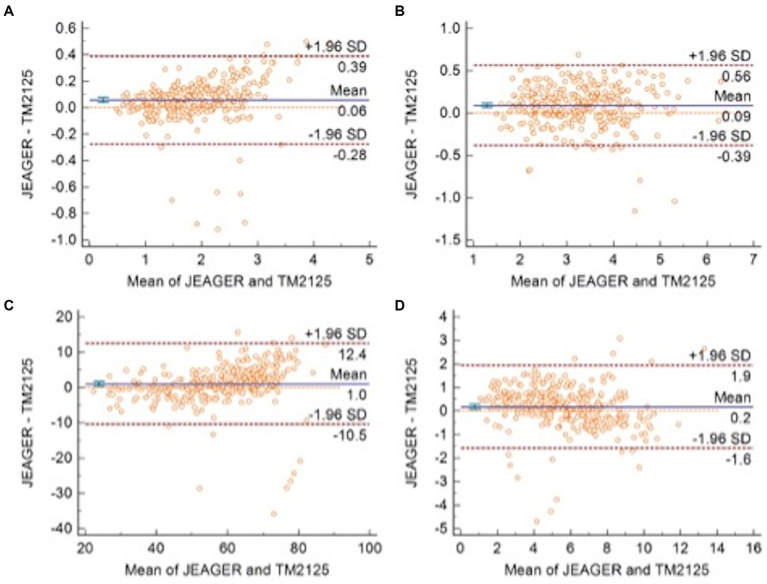
Bland–Altman plots with 95% Limits of Agreement of **(A)** FEV1, **(B)** FVC, **(C)** FEV1/FVC, and **(D)** PEF.

A total of 301 patients underwent spirometry using SUNVOU TM2125 and Jaeger MasterScreen. To evaluate the agreement and concordance between the two devices, we calculated the Pearson correlation and ICC for FEV1, FVC, FEV1/FVC%, and PEF (Table 2). Both metrics were quite high (greater than 0.9). The correlation plots for the parameters are presented in Figure 2, which showed good correlations between the parameters. To further evaluate the agreement between the measurements, we constructed a Bland–Altman plot (Figure 3). The plots showed a high degree of agreement, with mean differences of 0.06 L (95% CI:-0.28–0.39), 0.09 L (−0.39–0.56), 1.0% (−10.5–12.4), and 0.2 L/s (−1.6–1.9) for FEV1, FVC, FEV1/FVC%, and PEF.

**Table 2:** Comparison of measurements between SUNVOU TM2125 and Jaeger MasterScreen.

Parameters	JEAGER Masterscreen	SUNVOU TM2125	Differences:Jeager-TM2125	Paired T-test	Correlation	ICC
FEV1 (L)	1.93 ± 0.73	1.87 ± 0.69	0.06 ± 0.17	0.000	0.973 (*p* < 0.001)	0.971 (*p* < 0.001)
FVC (L)	3.31 ± 0.90	3.22 ± 0.90	0.09 ± 0.24	0.000	0.964 (*p* < 0.001)	0.964 (*p* < 0.001)
FEV1/FVC (%)	58.80 ± 14.08	57.83 ± 12.79	0.97 ± 5.84	0.004	0.910 (*p* < 0.001)	0.906 (*p* < 0.001)
PEF (L/s)	5.70 ± 2.28	5.53 ± 2.45	0.17 ± 0.90	0.001	0.930 (*p* < 0.001)	0.927 (*p* < 0.001)

## 4. Discussion

Spirometry and FeNO measurement are widely used for the diagnosis and management of respiratory diseases. These are the primary means of assessing respiratory function and inflammation (1, 2, 5). SUNVOU TM2125 can measure both spirometry and FeNO, which is easy to perform and provides a more cost-effective choice for primary hospitals. To confirm the clinical value of the “all-in-one machine,” we compared spirometric parameters (FEV1, FVC, FEV1/FVC%, and PEF) and FeNO values with two commonly used devices—Jaeger Masterscreen and NIOX VERO. According to the test results, SUNVOU TM2125 had a good correlation and high degree of agreement with the other devices, and there were no statistically significant differences.

The ICC values were all above 0.9, and the ICC of the four spirometric parameters were all above 0.9, which means that more than 90% of the differences in measurements came from individual differences between the subjects rather than systematic errors between the two devices. The differences (MasterScreen—TM2125) of FEV1, FVC, FEV1/FVC%, and PEF were 0.06 L (95% CI, −0.28 to 0.39), 0.09 L (−0.39 to 0.56), 1.0% (−10.5 to 12.4), and 0.2 L/s (−1.6 to 1.9) respectively. According to the standardization of spirometry, the repeatability validation limit for FEV1 and FVC was ±3.5% or ± 0.1 L, and the limit for PEF was ±12% or ± 25 L/min (10). The results were within this range. Therefore, the difference between the two spirometers was not statistically significant. The sequence of measurements performed by the devices may also cause differences. In our research, participants first took measurements with the MasterScreen, followed by TM2125. The interval between the two measurements was less than 20 min. Participants may experience fatigue due to forced expiration in the first measurement, which could affect the expiratory force of the second measurement. Many clinical studies have compared two spirometers, including portable spirometers, and the results of this study were similar to those studies (11–14).

There was a high correlation between the FeNO values measured using SUNVOU TM2125 and NIOX VERO (*r* = 0.988). According to the Bland–Altman plots, 93.2% of the values were within the 95% CI, indicating a high degree of agreement. The mean value of TM2125 was 1.8 ppb smaller than the mean value of VERO, which was lower than that reported in previous research. Many studies have compared chemiluminescence and electrochemical analyzers; the values of chemiluminescence analyzers were usually higher than those of electrochemical analyzers. The differences ranged from 3.3 to 9.4 ppb (15, 16). A comparison between electrochemical analyzers showed much smaller differences, ranging from 1.2 to 4.6 ppb (17, 18). The results of the present study are consistent with those of previous studies. Differences between the same types of analyzers could be caused by individual states. According to the guidelines published by the ATS in 2011, clinically significant changes in FeNO levels were > 10 ppb (or 20%). So, the differences of <10 ppb for measured FeNO values of <50 ppb and of <20% for FeNO values ⩾50 ppb were considered acceptable.

## Data availability statement

The raw data supporting the conclusions of this article will be made available by the authors, without undue reservation.

## Ethics statement

The studies involving humans were approved by China-Japan Friendship Hospital Review Board. The studies were conducted in accordance with the local legislation and institutional requirements. The participants provided their written informed consent to participate in this study.

## Author contributions

YL, KH, and TY contributed to the conception and design of the study and carried out data analysis and interpretation. TY provided administrative support. YL and TY provided study materials and patients. YL, KH, WL, YP, and XT collected and assembled data. YL, KH, WL, YP, XT, and TY contributed to writing and final approval of the manuscript. All authors contributed to the article and approved the submitted version.

## Funding

This study was supported by the CAMS Innovation Fund for Medical Sciences (2022-I2M-C&T-B-107 and 2021-I2M-1-049).

## Conflict of interest

YL, KH, WL, YP, XT, and TY have completed the ICMJE uniform disclosure form.

## Publisher’s note

All claims expressed in this article are solely those of the authors and do not necessarily represent those of their affiliated organizations, or those of the publisher, the editors and the reviewers. Any product that may be evaluated in this article, or claim that may be made by its manufacturer, is not guaranteed or endorsed by the publisher.
