# Supplementary material for: Validation of a new portable system containing both FeNO analysis and spirometry measurement
Source: Front Med (Lausanne). 2023 Aug 31;10:1210329. doi: 10.3389/fmed.2023.1210329 (PMC10501136; doi:10.3389/fmed.2023.1210329)
Supplement: Supplementary file 1 [file Data_Sheet_1.docx]

Supplementary materials for

Validation of a new portable system containing both FeNO analysis and spirometry measurement

Yong Li^1,*^, Ke Huang^1,*^, Wei Li^1^, Yaodie Peng^2^, Xingyao Tang^3^, Ting Yang^1,#^

Picture of the devices in this study


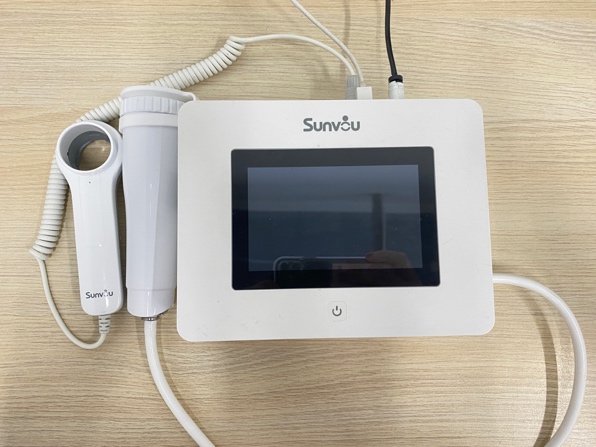


SUNVOU TM2125


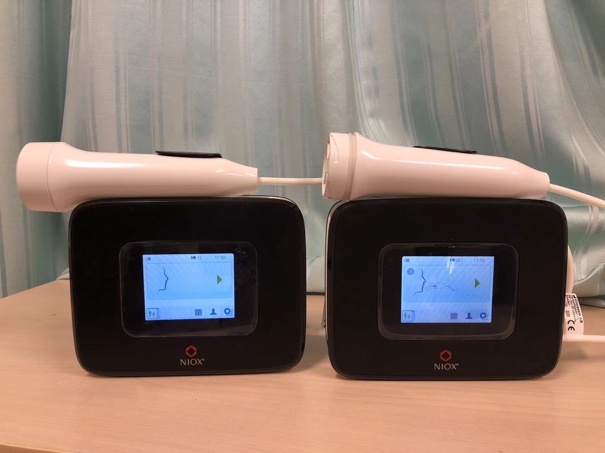


NIOX VERO


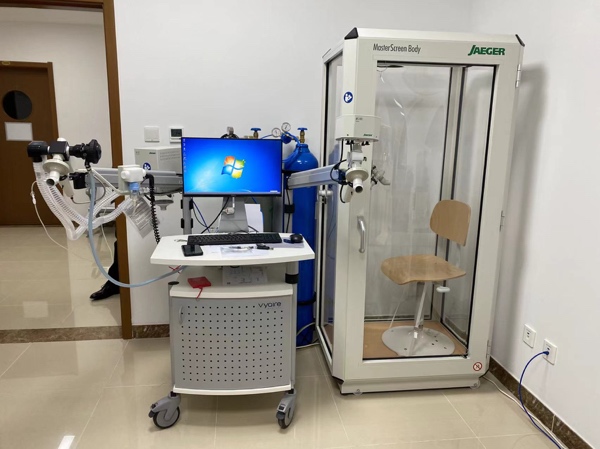


Jaeger MasterScreen
